# The Gut Fungus *Basidiobolus ranarum* Has a Large Genome and Different Copy Numbers of Putatively Functionally Redundant Elongation Factor Genes

**DOI:** 10.1371/journal.pone.0031268

**Published:** 2012-02-17

**Authors:** Daniel A. Henk, Matthew C. Fisher

**Affiliations:** The Department of Infectious Disease Epidemiology, Imperial College London, London, United Kingdom; University of California Riverside, United States of America

## Abstract

Fungal genomes range in size from 2.3 Mb for the microsporidian *Encephalitozoon intestinalis* up to 8000 Mb for *Entomophaga aulicae*, with a mean genome size of 37 Mb. *Basidiobolus*, a common inhabitant of vertebrate guts, is distantly related to all other fungi, and is unique in possessing both EF-1α and EFL genes. Using DNA sequencing and a quantitative PCR approach, we estimated a haploid genome size for *Basidiobolus* at 350 Mb. However, based on allelic variation, the nuclear genome is at least diploid, leading us to believe that the final genome size is at least 700 Mb. We also found that EFL was in three times the copy number of its putatively functionally overlapping paralog EF-1α. This suggests that gene or genome duplication may be an important feature of *B. ranarum* evolution, and also suggests that *B. ranarum* may have mechanisms in place that favor the preservation of functionally overlapping genes.

## Introduction

*Basidiobolus ranarum* is an occasional human pathogen found often in soil but most commonly detected in vertebrate guts and dung especially of amphibians and reptiles [1], [2], [3], [4], [5], [6], [7]. The phylogenetic position of *Basidiobolus* is not well resolved, but in most phylogenies it occupies a place somewhere between chytrid and Entomophthoromycotina lineages making it among the oldest lineages in the Kingdom Fungi [8], [9], [10]. *Basidiobolus* has numerous unique features that set it apart from other eukaryotes. Like many fungi *Basidiobolus* can grow as hyphae and/or yeast depending upon the environmental conditions, but when growing as hyphae the cytoplasm migrates with the growing tip making a mycelium of mostly empty cells and a collection of individual growing tip cells with no internal sharing of resources or genetic material. *Basidiobolus* has extremely large nuclei (25–50 µm) with nucleoli that can fill nearly the entire nucleus and a unique nuclear associated body superficially like a centriole but having a completely different microtubule arrangement from the canonical 9×3 microtubule arrangement in other eukaryotes.

*Basidiobolus* is also unique in its translation machinery. The gene EF-1α provides a core function of translation elongation by delivering t-RNA to the ribosome. Although EF-1α was initially believed to be a universal component of eukaryotic cells, it has relatively recently been discovered that some eukaryotes lack EF-1α and instead use a distantly related paralogous GTPase, Elongation Factor-Like (EFL), for translation and no longer have EF-1α [11]. The phylogenetic distribution of EFL is patchy across mostly single-celled eukaryotic lineages and both horizontal-gene-transfer and differential gene loss have been invoked to explain the distribution of EFL and EF-1α [12], [13], [14], [15]. Only *B. ranarum* and the marine diatom *Thalassiosira pseudonana* have been found to harbor both EF-1α and its distantly related paralog EFL with all other lineages having lost one or the other gene [8], [16]. There are currently no explanations for why *B. ranarum* has retained both EFL and EF-1α. In order to begin to better understand the evolution of *B. ranarum* we sought to determine the genome size of *B. ranarum* and the relative copy number of EFL and EF-1α genes using qPCR.

## Analysis

**Figure 1:**
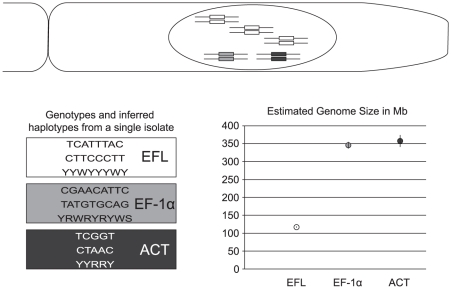
Schematic of *Basidiobolus* genome based on DNA sequence and qPCR. A) The large nucleus of *Basidiobolus* found in the growing tip cell of hyphal colonies is inferred to be larger than 700 Mb with multiple copies of each gene with three times as many copies of EF-1α as actin or EFL genes. B) Sequences recovered from allele specific PCR reconstitute genotypes from nonspecific PCR. C) Haploid genome size inferred from each gene shows the three-fold difference in inferred genome size of EFL compared to actin and EF-α consistent with a difference in copy number.

We sequenced three protein-coding loci, actin, EFL and EF-1α, from three strains of *B. ranarum* isolated from the dung of the amphibian *Discoglossus sardus*, collected on the island of Sardinia in the Mediterranean with permission from the Ente Foreste della Sardegna. These dung collections from a species not CITES listed do not require any special permission. Strains were isolated via the protocol of Nelson et al. (2002) and deposited in the USDA-ARSEF strain collection (Strain designations 9900, 9901, 9902). Sequences were deposited in GenBank with accession numbers JN817948-JN817965. We detected polymorphism for all loci within isolates based on ‘double peaks’ within the chromatograms suggesting that *B. ranarum* contains a base copy number of genes greater than one. In each locus, we detected within isolate polymorphism, with up to 6 polymorphic sites in actin, 7 in EFL, and 5 in EF-1α, and across all loci we found 18, 12, and 5 sites that were polymorphic within isolates 9900, 9901, and 9902 respectively. To confirm the nature of the within-isolate polymorphism we designed two primers to be identical except for the final 3′ base that covered a polymorphic position. This ensured that we would only amplify one of the two alleles, thus allowing the polymorphic site to be scored (Table 1). For all standard PCR we used Qiagen TopTaq kits with the same cycling conditions for each primer set, namely 95° for 15 minutes to activate the polymerase followed by 37 cycles of 95° for 30 seconds, a ‘touchdown’ annealing temperature starting at 60° for 30 seconds and decreasing one degree each cycle for the first 7 cycles to 54° for 30 seconds for each remaining cycle followed by 72° for 60 seconds. Each PCR with specific primers was successful, and sequencing of these products showed no double peaks but recovered all polymorphic sites in two haplotypes (Fig. 1).

**Table 1:** Primers and amplicons of PCR and qPCR.

Locus	PCR Primers 5′–3′ (amplicon length)	Quantitative PCR primers (amplicon length)
**Actin**	BactForward ACCACTGGTATCGTTCTTGACTCT	QBactForward GACTCTGGTGATGGTGTTACCCA
	BactReverse GACAGAGTATTTACGTTCAGG (550)	QBactReverse AGCGGTGGTGGTGAAAGTGTA (152)
	Allele specific BASactReverse ATTTACGTTCAGGAGGAGCA	
	Allele specific BASactReverse ATTTACGTTCAGGAGGAGCG	
**EF-like**	BeflForward CAAGAACATGATTTCCGGAGC	QBactReverse ATGGATTCCGATACCGCTGGT
	BeflReverse CGCATAGCAGCTTCGGTGTT (490)	QBeflReverse TCCTTCTTCCATCCGACCTTGA (92)
	Allele specific BASeflForward GCTGATGTTGCCCTTCTCATGGTT	
	Allele specific BASeflForward GCTGATGTTGCCCTTCTCATGGTC	
	BASeflForward GCTGATGTTGCCCTTCTCATGGTC	
**EF1-**	Bef1aForward GAAAAGGAAGCCGCTGAGCT	QBef1aForward CCCGACTCCAAGATAAGCCACT
	Bef1aReverse AAGATTGAAGCCTACAATGTCAC (700)	QBef1aReverse CACGACCAACAGGAACCGT (83)
	Allele specific BASef1aForward1 ATGCGCCCGGTCACCGAGACTTTATC	
	Allele specific BASef1aForward1 ATGCGCCCGGTCACCGAGACTTTATT	

To estimate the genome size of *B. ranarum* we used a quantitative PCR based approach outlined by Wilhelm et al. [17] and the following formula taken from Armaleo and May [18]: (Q_G_/Q_P_) * L_P_ * E^(CtG-CtP)^ = L_G_. Here, Q_G_ and Q_P_ are the starting quantities of DNA used in the qPCR reaction of genomic DNA and PCR-produced DNA, respectively, L_G_ and L_P_ are the lengths of the genomic and PCR-produced DNA, respectively, CtG and CtP are the quantitative qPCR Ct values of the genomic and PCR-produced DNA reactions, and E is the efficiency of the quantitative PCR. Based on this equation the inferred length of the genomic DNA should represent the haploid genome size, and if inferred lengths differ between loci then it would suggest either locus specific errors or copy number variation between loci. All things being equal, the inferred genome size should scale linearly with the copy number of the locus so that a single copy locus from a 10 Mb genome would estimate a genome of 10 Mb while a two-copy locus would estimate a genome of 5 Mb. We extracted genomic DNA using DNeasy Plant minikits (Qiagen) and used a Qubit fluorometer (Invitrogen) with the dsDNA BR kit to quantify total DNA from 10 µl aliquots of genomic DNA and PCR products, respectively. For qPCR, we used the QuantiTect SYBR Green PCR Kit (Qiagen) and a standard cycling protocol recommended by the kit manual of 95°C for 15 minutes followed by 35 cycles of 95° for 15 seconds, 60° for 30 seconds and 72° for 30 seconds data was acquired at the end of the extension step and during a final dissociation step moving from 60–90°. We used the following dilutions of PCR products as target DNA for each of the three loci independently: 10^−4^, 10^−6^, 10^−7^, 10^−9^. For genomic DNA we used undiluted and 10^−1^ dilutions as targets. We designed primers for qPCR that covered only sites that were not detected as polymorphic and amplified targets of 152, 98, and 83 bp of actin, EFL, and EF-1α loci, respectively. PCR efficiencies were calculated using the mean slope (each with r^2^ greater than 0.99) of the linear function of Ct values for the log dilution series of the PCR product qPCR reactions. This resulted in efficiencies of 1.96, 1.98, and 1.96 for actin, EFL, and EF-1α respectively. The average haploid genome size estimated from actin and EF-1α was 350 Mb while the average genome size estimated from EFL was 116 Mb. This suggests that the EFL locus is present in three times the copy number of the other two loci (Fig. 1).

## Results and Discussion

The average size of haploid fungal genomes is ∼37 Mb [19]. Therefore, this estimate of the *B. ranarum* genome is about ten times larger than the average fungus, but it is similar to several biotrophic fungal pathogens and still more than 20 times smaller than the *Entomophaga* genome estimated at 8000 Mb [20]. However, the haploid genome may be larger than the estimated 350 Mb if the actin and EF-1α genes are duplicated rather than in higher ploidy. Previous microscopic studies of mitosis have found numerous small chromosomes with estimates ranging from about 60 to many hundreds [21], [22]. Some chromosomes appear to be entirely within the nucleolus, and the mitotic process does not match any other fungi or indeed any other known organisms for that matter [1], [23]. It is possible that careful fluorescence microscopy and allele specific *in situ* hybridization in cells from multiple life stages of the fungus including zygospores could go some way towards distinguishing ploidy from copy number or genome duplication. The surprising result that EFL is apparently in three times the copy number of EF-1α suggests that gene or genome duplication may be an important feature of *B. ranarum* evolution. It also suggests that *B. ranarum* may have mechanisms in place that favor the preservation of functionally overlapping genes. The presence of both EFL and EF-1α in a single organism is very rare, having only been found for one other organism, the marine diatom *Thalassiosira pseudonana* [16]. This observation has been explained by a functional replacement of one paralog by another followed by gene-loss of the nonfunctional locus. However, if producing more of a gene is advantageous, it can favor the preservation of gene duplicates via gene conservation [24], and the same selective force acting to preserve the distant paralogs of EFL and EF-1α would act similarly to preserve multiple EFL copies. Diploidy is supported by the lifecycle of *Basidiobolus* and presence of two divergent haplotypes in actin and EF-1α because these genes had similar copy numbers detected via qPCR. However, chromosome copy number may also explain the increased copy number of EFL, making that region of the genome hexaploid. An increase in the base chromosome copy number can favor the accumulation of duplicate genes and simultaneously increase the recombination rate under sexual reproduction [25]. However, little is known about sexual recombination and ploidy in fungi outside of the well-studied dikaryotic lineages, and where it has been studied it is often complex [26]. Although zygospores are formed by *Basidiobolus*, meiosis has never been documented in *Basidiobolus* or any entomophthoralean fungus [27], [28]. The differing gene or chromosome copy numbers could also be generated and maintained by parasexual processes, which may fit the microscopic observations at least as well if not better than meiosis. Parasexual processes and variation in ploidy have been linked in *Candida albicans*, which has been found to maintain a nearly diploid genome but frequently undergo rounds of partial chromosome loss [26], [29]. Whether it makes sense to think of *Basidiobolus* as any particular ploidy may depend on making whole genome scale observations. If the presumed sexual stage is meiotic then *Basidiobolus* is likely to be both diploid and undergoing frequent sexual recombination, albeit entirely selfing. A population genetic study using these polymorphic genetic markers might distinguish between strict asexual propagation, parasexual recombination, selfing, and outcrossing in *Basidiobolus*, and would be a beneficial step towards better understanding the roles of ploidy and recombination in contributing to gene duplications and increasing genome size.
